# Neuromodulator and Emotion Biomarker for Stress Induced Mental Disorders

**DOI:** 10.1155/2016/2609128

**Published:** 2016-03-14

**Authors:** Simeng Gu, Wei Wang, Fushun Wang, Jason H. Huang

**Affiliations:** ^1^School of Psychology, Nanjing University of Chinese Medicine, Nanjing 210023, China; ^2^School of Psychology, Nanjing Normal University, Nanjing 210023, China; ^3^Department of Surgery, University of Rochester, Rochester, NY 14623, USA; ^4^Department of Neurosurgery, Baylor Scott & White Health, Temple, TX 76508, USA; ^5^Department of Surgery, Texas A&M College of Medicine, Temple, TX 76504, USA

## Abstract

Affective disorders are a leading cause of disabilities worldwide, and the etiology of these many affective disorders such as depression and posttraumatic stress disorder is due to hormone changes, which includes hypothalamus-pituitary-adrenal axis in the peripheral nervous system and neuromodulators in the central nervous system. Consistent with pharmacological studies indicating that medical treatment acts by increasing the concentration of catecholamine, the locus coeruleus (LC)/norepinephrine (NE) system is regarded as a critical part of the central “stress circuitry,” whose major function is to induce “fight or flight” behavior and fear and anger emotion. Despite the intensive studies, there is still controversy about NE with fear and anger. For example, the rats with LC ablation were more reluctant to leave a familiar place and took longer to consume the food pellets in an unfamiliar place (neophobia, i.e., fear in response to novelty). The reason for this discrepancy might be that NE is not only for flight (fear), but also for fight (anger). Here, we try to review recent literatures about NE with stress induced emotions and their relations with mental disorders. We propose that stress induced NE release can induce both fear and anger. “Adrenaline rush or norepinephrine rush” and fear and anger emotion might act as biomarkers for mental disorders.

## 1. Introduction

Affective disorders are a leading cause of disabilities worldwide [1], and the etiology of these many affective disorders such as depression and posttraumatic stress disorder (PTSD) is due to hormone changes, which includes hypothalamus-pituitary-adrenal (HPA) axis in the peripheral nervous system and neuromodulators in the central nervous system. The hypothalamic-pituitary-adrenal (HPA) axis plays a pivotal role in stress induced physiological changes [2], so that the levels of HPA axis, such as cortisol, are a good biomarker for certain mental disorders. In the central nervous system, neuromodulators, such as norepinephrine (NE), are regarded as a critical part of the central “stress circuitry” [3], which is consistent with pharmacological studies indicating that medical treatment acts by increasing the concentration of catecholamine. HPA axis and NE both can interact and interfere with each other; for example, NE release in the central nervous system facilitates activation of the hypothalamic-pituitary-adrenal axis in response to acute stress [4]. The major function of LC/NE system is to induce “fight or flight” behavior [5] and possibly fear and anger emotions. Hormonal hypothalamus-pituitary-adrenal (HPA) stress axis and the peripheral sympathoadrenal autonomic response system are possibly very good biomarkers for the physiological stresses, while the activities of the locus coeruleus (LC) or NE levels in the central nervous system are very good biomarkers for the mental stress.

## 2. NE and Stress

Stress can be defined as any threat, either real or perceived, to the homeostasis and wellbeing of an organism. Stress can be induced by two broad and qualitatively differentiated categories of stressors: physiological stress and psychological stressors. Physiologic stress is a physical threat to the wellbeing, and the psychogenic stress is the cognitive processing or interpretation of the stimulus as stressful [5]. The first category is a real and imminent physiological threat to health and wellbeing, and the second psychogenic stressor is cognitive interpretation of the stimulus as a stressful event. The locus coeruleus (LC) neurons can be activated by both stressful events, which means that information from both the external and the internal environment can activate LC/NE system [6]. Many electrophysiological and neurochemical studies have shown that the brain NE system is physically and robustly activated by a diverse array of acutely stressful stimuli [5].

The locus coeruleus (LC) is regarded as a part of the central “stress circuitry” [6, 7], because robust activation of the LC was reported after stressful stimuli [8]. The LC is the largest norepinephrine (NE) in the brain and projects axons to almost all brain regions. It is a pure NE nucleus in rodents, comprising approximately 1500 cells on each side of the brain stem in rats [9]. The neural substrates for the stress induced LC activation have not yet been clarified. The HPA axis is regulated by a number of neural and hormonal inputs to the hypothalamus, and noradrenergic system has been implicated as one of the important systems for the HPA stress axis, mainly through its action upon the corticotrophin-releasing factor neurons in the paraventricular nucleus (PVN) of the hypothalamus [10]. The NE afferents to the PVN originate mainly from the medullary NE nuclei and reach the PVN via the ventral NE bundle [6]. The CRF neurons in the PVN play the most important role in handling neuroendocrine stress response. The PVN contain neurons producing vasopressin and oxytocin, also regarded as hormones to cope with stressors, as well as regulating the water and electrolytes balance and parturition, respectively. The functional significance of this pathway in mediating stress responses was demonstrated in many studies, such as lesion of NA pathway [4]. Microinjection of NE directly into the PVN of conscious rats increases CRF gene expression in the PVN in parallel with ACTH release from the pituitary. The NE influence upon the PVN CRF neurons may be mediated by alpha-1-adrenergic receptors [4].

Stress induced activation of NE neurotransmission facilitates behavioral responses evoked by acute stress [9, 11], whether these responses represented inhibition of ongoing behavior, consistent with the behavioral function of LC/NE to say “no” [12], or activation of behavior that would not otherwise occur in the absence of acute stress. An inability to appropriately initiate or regulate aspects of the stress response has been proposed as a potentially critical factor in the pathophysiology of various stress-related disorders. The LC has been implicated in a variety of physiological functions, such as fear and anxiety [11]. Multiple brain regions have been implicated in emotional processing including anxiety and fear, the amygdala, the LC, the septohippocampal system, and the raphe nuclei. Consistently, the noradrenergic system in the brain is considered to play an important role in emotion, and central responses to stress, and has also been implicated in affective disorders. A considerable amount of evidence has suggested the relationship between the central NE and fear/anxiety [6]. Emotions are one possible means by which stress responses may be tailored to specific stress. NE is especially important to conditioned fear because fear learning occurs during times of threat and stress. NE release modulates the neuronal substrates of fear [13].

## 3. NE and Emotions

Stresses are known to activate norepinephrine (NE) release in the brain [8]. Dysregulation of LC/NE systems has been implicated in stress and its related psychiatric diseases such as depression, posttraumatic stress disorder (PTSD), and other anxiety disorders [6]. This notion is supported by the knowledge of the pharmacological actions of many psychotherapeutic drugs, including tricyclic antidepressants and noradrenaline reuptake inhibitors. The central noradrenergic system is also involved in panic disorders, especially posttraumatic stress disorders, because an alpha-2-adrenergic agonist, clonidine, is effective in alleviating the symptoms of patients with panic disorders [6]. NE release modulates the neuronal substrates of fear [13]. It was more than 30 years ago that the functional relationship of the LC with fear or anger was first suggested in monkeys by Redmond et al.: electrical stimulation of the LC resulted in particular behaviors that were observed in fearful or threatening situations in the wild [14]. NE is especially important to conditioned fear because fear learning occurs during times of threat and stress. Thereafter, considerable effort was made to examine whether the ablation of the LC of rodents elicits the behaviors consistent with those in monkeys [15].

However, the rats with 6-OHDA induced LC ablation were more reluctant to leave a familiar place and took longer to consume the food pellets in an unfamiliar place, suggesting an increase in fear following the lesion [9]. This is exactly opposite to the predictions of the fear hypothesis derived from monkey studies. Similarly, increase in neophobia (i.e., fear in response to novelty) was also observed in subsequent studies. The reason for this discrepancy between monkey and rat studies is not clear. It might be possible that NE has different controls over behaviors in different animals, for example, in prey and predators (anger in prey, fear in predator). For example, when a deer meets a lion, NE is released in the brains of both animals, but the reaction of the deer is flight (fear), while the reaction of a lion is fight (anger). So fear and anger are twin emotions coming from the same neuromodulator NE, but with different reaction depending upon the inner state of the animals [16].

## 4. Stress Induced Emotional Flows

*Fear is the path to the dark side.*

*Fear leads to anger.*

*Anger leads to hate.*

*Hate leads to suffering. (Master Yoda (George Lucas))*

Yoda was so wise and insightful that he said this. It is so true that you can test it in every situation when you are fearful; you will see it quickly turns into anger.

### 4.1. Emotional Flow

**Figure 1:**
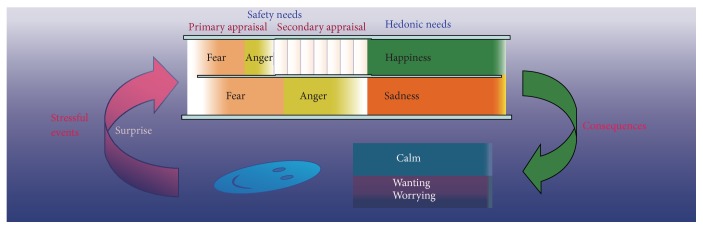
A cartoon showing the* emotional flow*. People first have a* safety* check for whatever things that may happen and get the fear and anger emotions if things happen unexpectedly (surprise), depending on available information. Fear is worrying about threats (primary appraisal). Anger comes after fear is gone when more information is available, and people are not scared anymore; instead, they started to blame the reasons for the unexpectancy (secondary appraisal). Fear and anger are not related to the* hedonic* characteristics of things, which are related to happiness or sadness. Usually, almost every stressful event can induce an* emotional flow*: fear-anger-happiness-sadness-calm (missing) (adapted from our previous paper [20]).

Everything in the world, whether it is positive or negative, occurs in ways expected or unexpected by us. Our ancestors negotiating rich environments were faced with a set of hugely complex inference and learning problems, involving many forms of variability [17]. Therefore, our ancestors evolved an adaptive mechanism to first have a safety check for everything in the world with available information, to see whether they are as expected or unanticipated. If something happens as anticipated, people feel calm; instead if it happens surprisingly, the first reaction of people would be to get scared and angry. This is an early evolutionary adaptation to allow better coping with dangerous and unexpected situations. Therefore, fear and anger* are due to unexpected ways in which these things happen* [16]. Let us take one example from Izard's paper; when Rafe was hit from the back by a wheelchair, the first reaction of him was to get scared and angry and he showed angry expression and clenched fist. But after he turned back to see Rebecca, a person with hemiplegia whose wheelchair had gone out of control and caused her to crash into Rafe, Rafe's understanding changed his anger to sadness and sympathy [18]. So when something happens, people will first evaluate whether it is dangerous (fear/anger) or not (calm) and next evaluate whether it fits into their need (happy/sad) (Figure 1).

Similar emotional flow happens all the time in our everyday life: life is normally calm; everything is as expected; then something unexpected happens; people first feel unsafe and scared (fear) and then blame (anger) the reasons for the unexpectancy after fear is gone; then people feel happy after successfully coping with the unsafe stimulation and/or feel sad if the individual failed to cope successfully. Finally, the stressful events go away, and people calm down. This kind of emotional wave, big or small, long or short, constitutes our everyday stressful emotional flow. So* fear-anger-happiness-sadness-calm* constitutes the* rainbow of emotions* or* emotional flow* in our everyday life (Figure 1).

### 4.2. Fear Leads to Anger

Even though fear and anger are the most frequently experienced emotions we have, there is no clear definition about them, let alone the relationship reports about them. From above, we can find that fear and anger are twin emotions that always come together at stressful events, usually fear first and anger next in tandem. For example, when an aggressive driver cuts in front of you, your first reaction is being scared (fear), and next you will feel angry after and only if fear is gone. So fear is the scariness from the unexpected coming of threat. Anger is the blaming of the reasons for the coming of unexpected things.

Similarly, Lazarus proposed a relational concept for stimulus and emotion: emotions are due to a relationship with the environment that the person regards as significant for his wellbeing [19]. Lazarus borrowed the concept of appraisal from Arnold and elaborated the concept as a key factor for emotions: emotional processes depend on the predictability of the stressful events. He distinguishes two basic forms of appraisal: primary and secondary appraisal [19], and he proposed that the primary appraisal and its induced emotions are faster activating, automatic processing, which is similar to the safety need. Indeed, Lazarus distinguished three types of stressful events: harm, threat, and challenge, which are related to primary appraisal. The secondary appraisal concerns coping options, which include blame or credit, coping potential, and future expectations. It seems that the primary appraisal is related to fear, and the secondary one is related to anger (Figure 1). Therefore, these two kinds of appraisals are related to safety needs: for personal safety, which are related to the “unexpected ways of stimulus occurring.” Evolutionarily, fear is related to threats, or aversive unconditional stimuli, such as pain and height. Due to our rational ability to think in the future and fantasize about “what might happen,” fear is getting broader in humans, such as being afraid of losing the needed things, in addition to getting the threat, because of the uncertainty/unexpectancy. Actually, the major reason is the uncertainty, because no matter what the hedonic value is, it has two sides of uncertainty, get it and lose it, which make us worry.

Anger is a nature passion aroused by a real or fancied injury or insult and involves a desire for retaliation. Anger always comes after fear at stressful events, and anger is due to fear, or more accurately, anger is due to the unexpected things, which induced fear [20].

### 4.3. Anger Is the Vent for Fear

Therefore, it seems that fear and anger are twin emotions that always come together, but fear and anger exclude each other in that they never occur at the same time in the same person. We give the equation below for the relationship between anger and fear, which fits in both duration and tension [20]:

(1) The  total  amount  of  Stress=fear  (duration,  tension)+anger  (duration,  tension).

Therefore, for a certain amount of stress, if fear is stronger, anger would be relatively less. For example, there is no time left for anger while riding a rollercoaster. On the contrary, when the fear is less, anger should dominate, such as betrayal by a lover. So it is thought that* anger has the function of suppressing fear*. At most of the time, fear and anger come in tandem with fear first and anger next. For example, after long time preparation for the test, Nancy was told to have failed in the test. She was scared at first, and then she was angry after she found out that it was not her fault, and her fear was gone for it was the teacher's mistake. Fear and anger are basic emotions, which are built into us to allow us to react to dangerous situations, just like in other animals. Yet the problems come because we have the rational ability to think in the future and past and fantasize about “what might happen.” So we might be angry, sometimes unperceived, because fear, most of which is based on subjective thinking, can quickly “overload” causing us to react in anger. So* anger is the vent for fear*. Anger is the secondary reaction to fear; if we are fearful, we will also have the tendency to be angry. Therefore, we have to keep our fears checked in order to control our anger.

### 4.4. Anger and Unexpectancy

Anger plays a significant role in everyday life [21], but there was no clear scientific definition about it before. If there is any, it would be very confusing; for example, anger is “an internal, mental, subjective feeling state with associated cognitions and physiological arousal patterns” [21]. Barries gave a simple definition: anger is the emotion humans experience when they do not get what they should or must get [22]; therefore, anger is due to unexpectancy.

It would be very useful to break down anger types, because anger takes many forms, such as bitterness, malice, clamor, envy, resentment, intolerance, criticism, revenge, wrath, hatred, sedition, jealousy, attack, gossip, sarcasm, and unforgiveness. Hughes divided anger into three types: the first form of anger, named “hasty and sudden anger” by Joseph Butler, is connected to the impulse for self-preservation. The second type of anger is named “settled and deliberate” anger and is a reaction to perceived deliberate harm of unfair treatment by others. These two forms are episodic. The third type of anger is dispositional and is related more to the character traits than to instincts or cognition. Irritability, sullenness, and churlishness postures are examples of the last form of anger [23]. One of the most common forms of breaking down anger into anger traits is habitual anger responses such as those determined by the State-Trait Anger Expression Inventory. This test addresses four different categories: trait anger, anger-out, anger-in, and anger control. Trait anger is loosely defined as the overall inclination towards anger. Anger-in itself refers more to the affective experience and is not to be confused with hostility or aggression. Anger-out implies that a person is more likely to deal with anger through outward expression such as verbal or physical behavior. The last factor, anger control, refers to the tendency to engage in behavior that is intended to reduce overt anger expression.

Anger is experienced more often than other emotions, and it is very intense and results in “adrenaline rush.” There are many people that have anger (fear) problems, and their problems are due to wrong beliefs. Our definition clearly states that anger is due to unexpectancy, so expectancy plays a key role in anger. So Ellis proposed that anger is not caused by the things that happen to us but is the result of wrong belief in our minds: we like to put the things “we would* like* to have,* wish* to have,* hope* to have, and* prefer* to having” into things “we BELIEVE we MUST have, SHOULD have, OUGHT to have, are OUTGHT TO have, and DEMAND to have* [sic]*” [24]. So in one sentence,* anger*/*fear is due to expectancy*,* or anticipation*, which depends on your belief.

## 5. Typical Mental Disorders

*Social Phobia*. In light of modern psychology, all diseases take their origin in anger. Anger pushes others away for people are often fearful of being exposed to angry people. Even the angry people themselves tend to isolate themselves from the world, because the angrier they get and the more the NE release, the more fearful they will be next time, and this results in social phobia. In the emotional flow, for the phobia patients, their problems might be that they cannot successfully accomplish the emotional flow (*fear-anger-happiness-sadness-calm*). The best way to remove fear is anger; these patients have problems with expression of anger, so their emotions are checked at emotional flowing from fear to anger.

*Depression*. Depression is characterized by unrelenting sadness accompanied by an inability to derive pleasure from positively hedonic situations. Therefore, depression might be related to the primary appraisal, the worrying about safety, instead of hedonic satisfaction. Indeed, excessive self-blame and feeling worthless are symptoms of major depression episodes across cultures [25], which is similar to Lazarus's secondary appraisal. So depressed patients have problems with anger or with coping appraisal, or their problem is due to inability to cope with the unsafe stressful situation, and they showed inward anger.

*Attention Deficit Hyperactivity Disorder (ADHD)*. ADHD is defined by persisting developmental inattention, impulsiveness, and hyperactivity. Recently, compelling evidence from genetic studies and pharmacological studies points to dysfunction of the central catecholaminergic network. Genetic analysis suggests that ADHD is caused by multiple genes, including dopamine receptors, the adrenergic receptors, and possibly also other monoamine transporters. Reduced levels of NE and DA have been demonstrated in rodent models of ADHD and in ADHD patients, consistent with pharmacological studies indicating that medical treatment acts by increasing the concentration of catecholamine.

## 6. Conclusions

A growing number of studies suggest that the activation of brain NE neurotransmission by acute stress acts to facilitate an array of neuroendocrine, autonomic, behavioral components of integrated, organismic response to stress. Given such a widespread modulatory effect on a number of adaptive responses to stress, it is possible that dysregulation of the brain NE system may represent a potential substrate for the stress-related psychopathology, such as depression, PTSD, or other anxiety disorders. Therefore, the neuromodulator levels in the cerebral spinal fluid and blood flow might act as biomarkers for stress induced mental disorders.
